# Synaptic Vesicle Recycling and the Endolysosomal System: A Reappraisal of Form and Function

**DOI:** 10.3389/fnsyn.2022.826098

**Published:** 2022-02-25

**Authors:** Daniela Ivanova, Michael A. Cousin

**Affiliations:** ^1^Centre for Discovery Brain Sciences, University of Edinburgh, Edinburgh, United Kingdom; ^2^Muir Maxwell Epilepsy Centre, University of Edinburgh, Edinburgh, United Kingdom; ^3^Simons Initiative for the Developing Brain, University of Edinburgh, Edinburgh, United Kingdom

**Keywords:** vesicle, endocytosis, endosome, trafficking, presynapse, lysosome

## Abstract

The endolysosomal system is present in all cell types. Within these cells, it performs a series of essential roles, such as trafficking and sorting of membrane cargo, intracellular signaling, control of metabolism and degradation. A specific compartment within central neurons, called the presynapse, mediates inter-neuronal communication via the fusion of neurotransmitter-containing synaptic vesicles (SVs). The localized recycling of SVs and their organization into functional pools is widely assumed to be a discrete mechanism, that only intersects with the endolysosomal system at specific points. However, evidence is emerging that molecules essential for endolysosomal function also have key roles within the SV life cycle, suggesting that they form a continuum rather than being isolated processes. In this review, we summarize the evidence for key endolysosomal molecules in SV recycling and propose an alternative model for membrane trafficking at the presynapse. This includes the hypotheses that endolysosomal intermediates represent specific functional SV pools, that sorting of cargo to SVs is mediated via the endolysosomal system and that manipulation of this process can result in both plastic changes to neurotransmitter release and pathophysiology via neurodegeneration.

## Introduction

Compartmentalization in eukaryotic cells enables efficient spatiotemporal control of multiple parallel cellular processes by concentrating the required factors in confined microenvironments that provide the best conditions for these processes to proceed. The endomembrane system, which includes the majority of membrane-bound organelles and the plasma membrane, plays a key role in segregating the intracellular environment into functional hubs. However, organelles do not operate as autonomous modules and without a regulated exchange of molecules between the compartments, their overall function would soon be compromised. Therefore, all membrane-bound organelles engage in extensive communication that coordinates their functions and enables long-term maintenance of cellular homeostasis.

### The Endolysosomal System

The endolysosomal system is a dynamic network of intracellular membranous organelles, where the endocytic, biosynthetic, and degradative pathways intersect (Maxfield and McGraw, 2004; Grant and Donaldson, 2009). This collection of organelles is a key sorting station distributing cargo to different membrane domains, a signaling hub regulating cellular metabolism and an intermediate to degradation (Klumperman and Raposo, 2014; Naslavsky and Caplan, 2018). The endolysosomal system has been extensively studied in non-neuronal cells, however, in neurons its characteristics remain relatively ambiguous and poorly defined (Andres-Alonso et al., 2021; Kuijpers et al., 2021a). In non-neuronal cells, the highly heterogeneous collection of organelles which constitutes the endolysosomal system is generally classified into several compartments, including early endosome (EE), recycling endosome (RE), late endosome (LE), and lysosome (Maxfield and McGraw, 2004; Klumperman and Raposo, 2014; Naslavsky and Caplan, 2018). It is still a matter of debate whether these heterogeneous compartments with overlapping, but also unique, set of proteins are distinct organelles or a series of structures that undergo maturation and “evolve” from each other.

The EE is a major station for sorting of cargo previously internalized by endocytosis. Some cargos are recycled via a rapid recycling pathway directly from EE, whereas others are trafficked to specialized RE, or endocytic recycling compartment, which is often but not always clustered in the perinuclear region, beside the microtubule-organizing center of the cell (Grant and Donaldson, 2009; Naslavsky and Caplan, 2018). In contrast to EEs, from which cargo can be recycled directly to the plasma membrane, the RE is involved in the “slow” recycling of internalized cargo (Li and DiFiglia, 2012). LEs are a group of organelles structurally and functionally related to lysosomes. Like lysosomes, LEs play a key role in protein degradation and are also essential components of the autophagy pathway (Scott et al., 2014). In addition, they are required for nutrient sensing and transport of cholesterol and other lipids to other membranous organelles in the cell (Scott et al., 2014). Lysosomes are the main waste disposal system of the cell, degrading materials delivered by autophagy or endocytosis into their basic building blocks. In addition, they participate in multiple other cellular processes, including regulation of metabolic signaling, gene expression, plasma membrane repair and lipid sensing and trafficking (Luzio et al., 2007; Saftig and Klumperman, 2009; Ballabio and Bonifacino, 2020).

### Synaptic Vesicle Recycling

The recycling of neurotransmitter-containing synaptic vesicles (SVs) at nerve terminals is one of the most extensively studied cellular pathways in central neurons. This process is essential for the neuronal communication, with even small perturbations resulting in a series of neurodevelopmental, neurodegenerative disorders or death (Bonnycastle et al., 2021; Overhoff et al., 2021). SVs are small 50 nm organelles that accumulate neurotransmitter via specialized transporters that are coupled to an intraluminal protonmotive force generated by V-type ATPases on the SV membrane. Neurotransmitter-filled SVs undergo a series of molecular events before they are triggered to fuse with the presynaptic plasma membrane during synaptic stimulation. These include a physical attachment to the plasma membrane (docking), a transition to fusion competency (priming), and calcium-triggered fusion (exocytosis) (Brunger et al., 2018; Rizo, 2018). After fusion, SV proteins and membrane are retrieved via endocytosis that is triggered by neuronal activity and proceeds at different speeds and locations in the nerve terminal (Chanaday et al., 2019).

Within central nerve terminals, three pools of SVs have been described based on their availability for release, the readily releasable pool (RRP), recycling pool and resting/reserve pool. A fourth pool, termed “super pool,” that is shared between neighboring *en passant* boutons and trafficked along the axon, has also been reported (Staras et al., 2010; Herzog et al., 2011; Zhang et al., 2019). Furthermore, several studies have suggested that separate SV pools drive different forms of neurotransmitter release (e.g., spontaneous, evoked synchronous and evoked asynchronous release) (Chanaday and Kavalali, 2018). However, this concept remains controversial, and has been refuted by others (Hua et al., 2010). This controversy may in large part be due to the fact that the majority of current studies focus on SV recycling as an isolated process. Our understanding of how the SV cycle integrates in the general endomembrane system in neurons and the functional implications of its communication with other membrane trafficking pathways, remains limited. Specifically, very little is known about the biogenesis and maintenance of the SV cluster across the lifespan of the neuron. Furthermore, fundamental questions about the mechanisms that mediate turnover of SV components also remain unanswered. These questions cannot be adequately addressed unless a more holistic understanding of the SV cycle and its place in the general endomembrane system in neurons is developed.

However, this important question is difficult to address, since there are only a few distinctive molecular markers that define EE and especially RE, which has made the characterization of their cellular localization and function a challenging endeavor. Defining EE and RE, their function in neuronal cells, and in particular in synaptic terminals, has proven to be a monumental task, partly due to the reductionist approach to SV recycling largely adopted by the field. Although it has its place, the fixation on detail often leads to inability to “see the wood for the trees.”

There is accumulating evidence to suggest that SV recycling and axonal endolysosomal trafficking are both structurally and functionally linked. However, the extent to which they are entangled is difficult to envision because of the lack of a conceptual framework that juxtaposes the two pathways in synaptic terminals. In this review, we propose an alternative interpretation of SV recycling by proposing that the SV pool is an integral part of the endolysosomal system in neurons, with equivalents of EE, RE, LE, and lysosomes functioning in concert at the presynapse, and supporting not only synaptic transmission, but the overall health of the neuron. In doing so, we will discuss the current evidence for crosstalk and potential overlap between the endolysosomal system and the SV recycling pathway, by examining the role of endolysosomal molecules in SV recycling which form the foundation of a series of testable hypotheses for future studies.

## Endocytosis and Early Endosomes

### Endocytosis

The first stage of the endolysosomal pathway is endocytosis. Due to its capacity to regulate the surface expression and internalization of membrane and soluble molecules, endocytosis is a fundamental cellular mechanism that regulates a multitude of cellular processes, including uptake of nutrients, cell signaling, establishment of cellular polarity, cell motility, and neurotransmission (Conner and Schmid, 2003; Doherty and McMahon, 2009). Although clathrin-dependent endocytosis is the most studied and best characterized endocytosis pathway, owing to their high capacity, clathrin-independent mechanisms are now recognized as the main route for internalization of cargo, responsible for the majority (∼70%) of membrane and fluid uptake into the cell (Howes et al., 2010; Renard and Boucrot, 2021). Neurons and synaptic terminals are no exception from this rule. There is accumulating evidence, that clathrin-independent, bulk membrane retrieval is also the dominant pathway for membrane retrieval in central nerve terminals across a range of stimuli, but particularly during intense neuronal activity (Clayton et al., 2008; Kononenko et al., 2014). During this endocytic process, known as activity-dependent bulk endocytosis (ADBE), endosomes are formed directly from the plasma membrane (bulk endosomes) with SVs subsequently regenerated from these compartments (Kokotos and Cousin, 2015). Clathrin still performs an essential role in the reformation of SVs and selection of SV cargo, however, there is an emerging consensus that this occurs at the level of the internalized endosome, rather than at the plasma membrane (Watanabe et al., 2014; Kononenko and Haucke, 2015). The formation of endosomes can occur at different timescale from tens of milliseconds (e.g., the recently reported ultrafast endocytosis) to tens of seconds, depending on the strength of the stimulus input (Watanabe et al., 2013, 2018; Soykan et al., 2017). The lack of clathrin dependence of SV endocytosis is particularly pertinent in experiments performed at physiological temperatures (Kononenko et al., 2014; Watanabe et al., 2014; Delvendahl et al., 2016). In these conditions, the relatively slow assembly of the clathrin coat means that clathrin-mediated budding events that originate on the plasma membrane are finalized on the endosome, due to the rapid invagination of these structures. Several other forms of endocytosis have been proposed to operate at the presynapse: e.g., clathrin-independent endocytosis (CIE) (Soykan et al., 2016), clathrin-independent and calcium-independent endocytosis (Orlando et al., 2019) and “kiss-and-run” (Stevens and Williams, 2000; He et al., 2006; Zhang et al., 2009). However, they either represent the same process of clathrin-independent bulk membrane retrieval under a different name (CIE) or their existence and contribution to presynaptic function at typical small central nerve terminals remains a matter of debate (“kiss-and-run”) (Granseth et al., 2006; LoGiudice and Matthews, 2006; Wu et al., 2014).

### Early Endosome Function

Classical EEs in non-neuronal cells are marked by a series of molecules with specific functional roles. For example, they typically contain a high concentration of the lipid phosphatidylinositol 3-phosphate (PI(3)P), generated by the PI3-kinase VPS34 (Grant and Donaldson, 2009). VPS34 is a class III phosphatidylinositol 3-kinase that uses phosphatidylinositol as a substrate to generate PI(3)P. Previous studies have revealed an important role for VPS34 in EE-sorting and autophagy (Dall’Armi et al., 2013; Raiborg et al., 2013). Accumulation of PI(3)P is required for the recruitment of proteins to EEs that contain a FYVE PI(3)P-binding domain, such as early endosomal antigen 1 (EEA1) and rabenosyn-5. EEA1 functions as a tethering molecule that together with the endosomal soluble N-ethylmaleimide attachment protein receptor (SNARE) proteins controls the maturation of EE and their homotypic fusion (Maxfield and McGraw, 2004). SNARE proteins are classified as either Q- or R-SNAREs dependent on the presence of highly conserved glutamine or arginine residues at the center of their SNARE motif that drives fusion (Jahn and Scheller, 2006). Typically, R-SNAREs reside on the vesicular membrane whereas Q-SNAREs reside on target membranes. Rabenosyn-5 is also required for early endosomal fusion. In addition, rabenosyn-5, associates with another important class of EE-associated proteins, the dynamin-like EHD (Eps15 Homology Domain) ATPases, to regulate the transport of cargo from EEs to REs (Naslavsky and Caplan, 2011).

Another layer of identity for the organelles in the endolysosomal system is provided by the small Rab GTPases. They are considered “deciphers of organelle identity” and serve as platforms for recruitment of specific molecular machineries that confer unique functional characteristics to the particular organelles they bind to Stenmark (2009). The Rab GTPases that typically associate with EEs include Rab4, Rab5, Rab10, Rab14, Rab21, and Rab22 (Stenmark, 2009). It has been suggested that Rab proteins on EE cluster in district membrane microdomains. However, the precise mechanisms by which proteins are clustered into these domains remain poorly understood.

The SNARE-based vesicle fusion system is key for endosomal fusion and transport. After cargo proteins have been sorted into EE subdomains, a process of budding and fission of tubulovesicular structures occurs and the newly formed vesicles are trafficked to their target organelle (Grant and Donaldson, 2009). Some of the most important early endosomal SNAREs involved in homotypic and heterotypic endosomal fusion include VAMP4, syntaxin 6, syntaxin 12 (called syntaxin 13 in rat), and Vti1a. The R-SNARE VAMP4 and the Q-SNAREs syntaxin 6, syntaxin 12, and Vti1a predominantly localize at the trans-Golgi network (TGN), but are also found at the plasma membrane, EE and RE, with VAMP4 and syntaxin 6 also present on lysosome-related secretory organelles (Maxfield and McGraw, 2004). All four are implicated in the homotypic fusion of EEs by forming a SNARE complex with each other. In addition, VAMP4 and Vti1a have a central role in the retrograde endosome-to- TGN transport (Johannes and Popoff, 2008; Hirata et al., 2015). This pathway is important for recycling of molecules to the TGN, that in turn enable efficient anterograde transport of transmembrane proteins. Both VAMP4 and Vti1a are involved in maintaining the ribbon structure of the TGN (Shitara et al., 2013).

### Early Endosome and Synaptic Vesicle Recycling

When considering the role of EEs in SV recycling, a key question to address is—are any of the molecules mentioned above implicated in this process? Many of the EE markers outlined above are enriched in nerve terminals and some have links to SV recycling.

In neuronal axons, PI3-kinase activity is important for long-range trafficking, actin-based membrane ruffling and bulk membrane retrieval in growth cones, all of which support axonal outgrowth during neuronal development (Bonanomi et al., 2008; Zhou et al., 2013; Lorenzo et al., 2014). In mature presynaptic boutons, PI3-kinase activity supports actin remodeling and a signaling cascade linked to ADBE (Holt et al., 2003; Nicholson-Fish et al., 2016). Furthermore, inhibition of PI3-kinase activity stalls SV recycling and results in the appearance of numerous cisternae in synaptic terminals (Rizzoli and Betz, 2002; Richards et al., 2004). Presynaptic PI3-kinase activity is coupled to membrane depolarization and calcium influx, in a similar manner to SV recycling (Nicholson-Fish et al., 2016). PI3-kinase activity is also associated with the SV protein synapsin and it is required for optimal replenishment of the RRP from the reserve pool (Cousin et al., 2003). However it should be noted that the PI3-kinase activity associated with synapsin was from Class I PI3-kinases [which generate PI(3,4,5)P_3_], whereas the activity required for classical EE function is via Class III [which generate PI(3)P]. Although, FYVE-domain-containing proteins, such as EEA1 and rabenosyn-5 were reported to show a polarized distribution to the somato-dendritic domain, they have been detected in axons (Selak et al., 2004; Ackermann et al., 2019; Goto-Silva et al., 2019). However, more research is warranted to shed light on their exact presynaptic function. Finally, biochemical studies examining endosomes purified from AP1 (adaptor protein 1) σ knockout mice suggest that VPS34 is required for the maturation of LE and lysosomes (multivesicular bodies) in axons via a mechanism which involves sorting at presynaptic endosomes mediated by ADP-ribosylation factor GTPase activating protein 1 and Rab5 GDP/GTP exchange factor (Candiello et al., 2016). This implies that, in presynaptic terminals, PI3-kinase activity may be at the core of a mechanism operating at the presynaptic endosomes which coordinates both generation of functional SVs and degradative pathways.

More than 11 distinct Rabs, including most of the early endosomal Rabs, were detected on the surface of highly purified SV membranes (Pavlos et al., 2010; Pavlos and Jahn, 2011). The best characterized early endosomal Rab, Rab5, is present in a subpopulation of SVs at the presynapse, with its manipulation resulting in alterations to SV recycling. For example, overexpression of Rab5 reduced the size of the recycling SV pool in hippocampal neurons by 50 % (Star et al., 2005), whereas dominant negative Rab5 expression impaired SV recycling (Shimizu et al., 2003; Wucherpfennig et al., 2003). Furthermore, wild-type and constitutively active Rab5 rescued defective SV endocytosis produced by knockdown of the protein kinase leucine rich repeat kinase 2 (LRRK2) (Shin et al., 2008). Another early endosome Rab, Rab4, traffics bidirectionally within the axon and is enriched at synapses, with a reduction in its anterograde trafficking resulting in aberrant synaptic morphology (Dey et al., 2017; White et al., 2020). Therefore, classical EE Rabs are present at the presynapse and modulate the SV life cycle, providing further evidence of crosstalk and functional integration.

A number of endosomal SNAREs are implicated in SV recycling. For example, a series of early endosome Q-SNARE proteins (Vti1a, syntaxin-6 and syntaxin-13) visit the presynaptic plasma membrane during brief stimulation or baseline activity, suggesting that endosomal SNAREs were present on SVs (Hoopmann et al., 2010; Ramirez et al., 2012). Vti1a resides on SVs that recycle at rest and sustain spontaneous neurotransmission (Ramirez et al., 2012). More recently, Vti1a (and Vti1b) were implicated in regulation of SV- and dense-core vesicle fusion at the presynapse by modulating secretory cargo sorting at the TGN (Emperador-Melero et al., 2018). The endosomal R-SNARE VAMP4, is present on highly purified SVs (Takamori et al., 2006), however, it does not readily visit the cell surface during neuronal activity (Raingo et al., 2012; Nicholson-Fish et al., 2015; Ivanova et al., 2021). VAMP4 has been proposed to control discrete forms of neurotransmission (Raingo et al., 2012; Lin et al., 2020), however, a more global role has recently been identified. This role is of a negative regulator of SV release probability (Pr), with VAMP4 levels on SVs controlled via both ADBE and downstream endolysosomal processing (Ivanova et al., 2021). Altogether, this highlights EE proteins as an integral component and a key modulator of SV recycling.

### Retromer Function

The retromer protein complex is a critical component of the machinery mediating sorting and trafficking from EE (Seaman, 2012; Burd and Cullen, 2014). It was first characterized in yeast as a complex involved in the retrograde trafficking of membrane proteins, from peripheral endosomes to the TGN (Seaman et al., 1998), but a role in the trafficking of cargo to the plasma membrane has also been described (Small and Petsko, 2015). It consists of two main parts, the cargo-selection complex (CSC) and the tubulation module. In mammals, the two modules are not tightly coupled and can function independently. The CSC is composed of three proteins VPS26, VPS29, and VPS35, whereas the tubulation module is a heterodimer of the BAR (Bin, Amphiphysin, Rvs)-containing sorting nexins SNX1/SNX2 and SNX5/SNX6 (Seaman, 2012; Gallon and Cullen, 2015).

It is assumed that the GTPase activity of dynamin is important for scission of retromer-containing vesicles at EEs (Naslavsky and Caplan, 2018). Association of EHD ATPases and several of their endocytic interaction partners (e.g., syndapin and MICAL-like protein 1) with retromer in domains where vesicles are being generated at EE has also been shown (Gokool et al., 2007; Naslavsky and Caplan, 2011; McKenzie et al., 2012). It was suggested that both motor pulling by different fission complexes and the force generated by the WASH-mediated nucleation of actin, are important for the budding of vesicles from EE (Derivery et al., 2009; Jia et al., 2012; Capitani and Baldari, 2021).

### Retromer and Synaptic Vesicle Recycling

The retromer complex and EHD are ubiquitously expressed in the nervous system, and they are both enriched in the presynaptic compartment (Jakobsson et al., 2011; Li and DiFiglia, 2012; Inoshita et al., 2017). Retromer function is essential for neuronal development and constitutive knockout of VPS35 is embryonically lethal, whereas heterozygous knockout hinders the development of both axons and dendrites (Wen et al., 2011; Tian et al., 2015). Investigation of the effect of VPS35 knockdown on presynaptic function in mouse hippocampal neurons, failed to detect any deficits in SV exo- and endocytosis (Vazquez-Sanchez et al., 2018). However, deletion of VPS35 in Drosophila larvae led to a reduced number and altered morphology of SVs in motor terminals which was accompanied by enhanced rundown of synaptic transmission, suggesting a functional role of retromer in SV recycling (Inoshita et al., 2017). However, given the apparent discrepancy between these two model systems, further studies are required to adjudicate on the precise function of retromer in the SV cycle. Interestingly, retromer dysfunction is involved in the pathoetiology of neurodegenerative diseases such as Alzheimer’s and Parkinson’s disease, a common early hallmark of which is disruption of presynaptic function (Small and Petsko, 2015).

EHD1 is also linked to SV recycling. In addition to a potential negative regulation of SV exocytosis (Wei et al., 2010), EHD1 is required for clathrin-independent endocytosis. For example, its removal at the lamprey giant reticulospinal synapse, blocks SV endocytosis due to defective dynamin-induced membrane tubulation (Jakobsson et al., 2011). Furthermore EHDs associate with syndapins (Braun et al., 2005), F-BAR-proteins with pivotal role in ADBE and SV reformation at bulk endosomes (Clayton et al., 2009; Koch D. et al., 2011; Quan et al., 2012; Cheung and Cousin, 2019).

## Recycling Endosome

### Recycling Endosome Function

An alternative mechanism for trafficking of cargo from the EE to the RE involves a process of organelle maturation. This mechanism does not require generation of vesicles from the EE and their fusion with the RE, but the entire EE changes its identity and matures to RE by acquiring a set of characteristic proteins, such as Rab11, Rab35, cellubrevin (VAMP3), ADP-ribosylation factor 6 (Arf6), and EHD1 (Grant and Donaldson, 2009). There are no typical resident proteins that define REs. Thus, rather than a “stable” compartment that receives cargo from the EE, the RE can be viewed as the residual organelle that remains from the EE after cargo sorting to LEs. As stated above, the major function of REs is as a trafficking intermediate for cargo proteins that normally undergo recycling to the plasma membrane. In most cell types, REs are a collection of tubule-vesicular structures of approximately 60 nm in diameter that localize to the perinuclear region in the vicinity of microtubule-organizing center (Li and DiFiglia, 2012; Goldenring, 2015). However, the perinuclear distribution of REs does not seem to be essential for their function and in some cells, REs are dispersed throughout the cytoplasm (Joensuu et al., 2017; Naslavsky and Caplan, 2018).

Rab11 has been broadly accepted as the main regulator of slow recycling through REs and its function is implicated in the recycling of a vast array of membrane proteins ranging from cell adhesion molecules to membrane receptors and ion channels (Li and DiFiglia, 2012). How Rab11 exerts its function in the regulation of RE trafficking is not well established, however. One prediction is that Rab11 controls the formation of vesicles from RE by recruiting EHD1 and its interactors, such as syndapin2, that collectively promote fission of cargo carriers (Naslavsky and Caplan, 2011; Li and DiFiglia, 2012). RE have been reported to also function as a sorting intermediate for proteins synthesized at the TGN that are destined for secretion (Murray et al., 2005). The integrity of the R-SNARE VAMP3/cellubrevin and Rab11 functions were shown to be crucial for the RE-mediated secretion of newly synthesized proteins (Murray et al., 2005).

The GTPase Arf6 is another molecular marker regulating the endocytic trafficking through RE whose function is essential in all cell types for multiple cellular events, including regulation of cell shape, cytokinesis, cell migration, and tumor cell invasion (Sheehan and Waites, 2019). Rab35 is also required for recycling previously endocytosed cargo to the plasma membrane as part of the RE system (Klinkert and Echard, 2016). Intricate reciprocal antagonistic processes regulate the active/inactive state of both Rab35 and Arf6, suggesting these GTPases are key effectors in RE function (Sheehan and Waites, 2019).

As stated above, a systematic investigation of RE has been challenging because of the difficulties identifying unique molecular tags for this organelle. The challenge to decipher the molecular basis of the RE points to the possibility that REs are not a single organelle but a constellation of interconnected, semi-autonomous organelles that collectively coordinate the trafficking of specific cargos to the plasma membrane. Therefore, it is easy to envisage that specializations of this system exist within different cell types and cellular compartments and account for regulation of the trafficking of compartment-specific cargo molecules.

### Recycling Endosomes and Synaptic Vesicle Recycling

There is accumulating evidence that key components of the RE machinery, such as Rab35, Arf6, and their effectors, perform important roles in both SV recycling and processing of SV cargo. For example, *Drosophila* hypomorphic mutants for the Rab35 GAP, *skywalker*, display a large increase in presynaptic endosomes during neuronal activity (Uytterhoeven et al., 2011). Constitutively active Rab5, 23, and 35 mutants all phenocopied this defect, suggesting a key role of EE/RE effectors in SV recycling. The human *skywalker* orthologue TBC1 Domain Family Member 24 (TBC1D24) appears to perform a parallel role in mammalian nerve terminals. For example, its depletion caused defective growth cone endocytosis and axonal initial segment maturation resulting in altered action potential firing (Aprile et al., 2019). In addition, neurons from mice haploinsufficient for TBC1D24 displayed dysfunctional SV endocytosis, with a threefold increase in the volume of presynaptic endosomes, consistent with the phenotype in *Drosophila sky* mutants (Finelli et al., 2019). Interestingly, similar SV recycling phenotypes are observed in neurons in which endogenous Arf6 was depleted (Tagliatti et al., 2016). Since TBC1D24 regulates the activation state of Arf6 (Falace et al., 2014; Aprile et al., 2019), this suggests that Arf6 represses trafficking via endosomal SV recycling routes, whereas Rab35 promotes endosomal recycling. In support, depletion of the Rab35 guanine nucleotide exchange factor connecdenn in primary neuronal culture greatly reduces endocytosis during strong stimulation (Allaire et al., 2006). In addition, the Rab35/Arf6 system controls the degradation of specific SV cargos. For example, increased endosomal flux via *skywalker* increases the functional size of the RRP, due to rejuvenation of specific SV cargo components (Uytterhoeven et al., 2011). Furthermore, interference with this endosomal sorting route restored RRP size (Fernandes et al., 2014).

Rab11 is associated with both REs and LE/lysosomes, and has also been linked to SV recycling, mainly in model organisms. In these studies, Rab11 performs a facilitatory role. For example, its knockdown in *C. elegans* resulted in SV endocytosis defects (Han et al., 2017), whereas its expression in either a *Drosophila* model of Huntington’s Disease or Vps35 null flies restored the SV size to control levels (Steinert et al., 2012; Inoshita et al., 2017). In mammalian neurons, overexpression of constitutively active Rab11 mutants facilitated SV endocytosis (Kokotos et al., 2018). Therefore, molecules essential for RE function are also required for optimal SV endocytosis and cargo trafficking at the presynapse. This suggests that REs are fully integrated into the SV recycling system and even that some SVs that undergo spontaneous and evoked fusion may in fact be considered as a type of REs.

## Late Endosomes/Lysosomes

### Late Endosome/Lysosome Function

Molecular discrimination between LEs and lysosomes is challenging because of the absence of selective molecular markers between these organelles. However, these compartments have different origins. LEs are formed from dynamic EEs as endocytic carrier vesicles, which undergo a conversion during which the small GTPase Rab5 is exchanged for Rab7 (Stoorvogel et al., 1991; Rink et al., 2005). Similar to other types of endosomes, they are a heterogeneous group of organelles. One specific kind of LE contains lumenal vesicles and are often described as multi-vesicular bodies (MVB) (Piper and Katzmann, 2007). The sorting of ubiquitinated membrane proteins into intralumenal vesicles and the mechanism by which MVB are formed, which typically involves the function of the ESCRT (endosomal sorting complexes required for transport) complexes, has been extensively discussed elsewhere (Hurley, 2008, 2015; Wollert and Hurley, 2010; Vietri et al., 2020).

Lysosomes on the other hand, are the terminal degradative compartment for cargo internalized through endocytosis and intracellular cargo segregated during autophagy. Lysosomes are formed from the TGN, in a process during which lysosomal transmembrane proteins are delivered directly to the lysosome, whereas newly synthesized acid hydrolases are transported to the lysosome through an endosomal intermediate (Saftig and Klumperman, 2009). The indirect route allows TGN recycling of the mannose-6-phosphate receptor, which binds to the mannose-6-phosphate tag on the acid hydrolases and sorts them to the lysosome. Lysosomes are, essentially, storage containers for degradative enzymes, which periodically fuse with late endosomes, autophagosomes, or other hybrid organelles (amphisomes), to form a compartment in which degradation occurs (Ballabio and Bonifacino, 2020). The regeneration of functional lysosomes from these compartments after degradation is another route for maintaining the lysosomal pool and cellular homeostasis (Yang and Wang, 2021). However, the function of lysosomes is not restricted to degradation of cellular components: they can also undergo regulated exocytosis in response to an increase in the intracellular calcium concentration (Martinez et al., 2000). Lysosome exocytosis is believed to supply extra membrane for plasma membrane repair (Reddy et al., 2001), which is an essential homeostatic mechanism that prevents cell death and progression of multiple diseases (Zhen et al., 2021).

Similar to other fusion events, fusion of lysosomes or late endosomes with the plasma membrane is a sequential process that proceeds through different stages: tethering, formation of a trans-SNARE complex and fusion (Luzio et al., 2007). Organelle tethering is a prerequisite step for fusion, during which membrane organelles form links with each other. The composition of the tethers responsible for LE-lysosome fusion has not been completely established, but homotypic fusion and protein sorting complex, which is recruited by Rab7, is likely one of the components (Fernandes et al., 2014). Following tethering, the formation of a SNARE complex bridges across the two membranes and enables fusion. Compelling evidence exists that the Q-SNAREs syntaxin-7, syntaxin-8, and Vti1b are essential for both, homotypic late endosome fusion and heterotypic late endosome-lysosome fusion, whereas the R-SNARE VAMP7 is specifically required for late endosome-lysosome fusion (Luzio et al., 2010). VAMP7 also mediates fusion of lysosomes with the plasma membrane (Rao et al., 2004). The N-terminal longin domain of VAMP7 is a critical regulatory site for its endosomal sorting. The kinetics and the extent of the calcium-dependent fusion of lysosomes with the plasma membrane are regulated by the calcium sensor synaptotagmin-7 (Syt7) (Martinez et al., 2000).

### Late Endosome/Lysosomes and Synaptic Vesicle Recycling

In neurons, LEs formed at the synapse undergo progressive acidification and further maturation toward a lysosomal identity during their retrograde transport to the cell body (Deinhardt et al., 2006; Maday et al., 2012; Cheng et al., 2015). In agreement with this model, organelles with increasing levels of acidity are observed from distal to proximal axons (Overly and Hollenbeck, 1996). The controversy surrounding the presence of *bone fide* lysosomes at the presynapse is compounded by the fact that common markers of lysosomes such as LAMP1 are present on many non-lysosomal compartments (Vukoja et al., 2018). However, it is clear that fusion of either LEs, lysosomes, or vesicles containing lysosome markers, occurs at distal axons and is both calcium- and SNARE-dependent, with both Syt7 acting as the calcium sensor and VAMP7 as the R-SNARE on the LE/lysosome membrane (Arantes and Andrews, 2006).

Evidence has been accumulating that both Syt7 and VAMP7 (and potentially by extension LEs) are integrated in the SV life cycle. VAMP7 is targeted to SVs via an interaction with its longin domain to the adaptor complex AP3 (Scheuber et al., 2006). Interestingly, the VAMP7 longin domain interferes with the formation of SNARE complexes with a variety of Q-SNARE partners (Martinez-Arca et al., 2003), providing a potential explanation for why VAMP7 fails to visit the cell surface during action potential stimulation (Hua et al., 2011; Ramirez et al., 2012). However, VAMP7-containing vesicles may support spontaneous SV fusion, since increased cell surface trafficking occurs in resting neurons. In support, expression of VAMP7 lacking its longin domain increases spontaneous SV fusion, suggesting that VAMP7 may be required for this event (Hua et al., 2011). Furthermore, modulation of spontaneous SV fusion events by the signaling molecule reelin was abolished on depletion of endogenous VAMP7 (Bal et al., 2013), suggesting that VAMP7-mediated fusion events occur at the presynapse and can be modulated.

Almost all of the proposed presynaptic functions for Syt7 are dependent on its role as a calcium sensor. For example, Syt7 is postulated to be the calcium sensor for store-operated channel entry-mediated presynaptic calcium increases from the endoplasmic reticulum (ER), which augment spontaneous glutamate release (Chanaday et al., 2021). In addition, Syt7 is proposed to replenish the RRP via a calcium-dependent interaction with calmodulin (Liu et al., 2014). Syt7 is also a candidate calcium sensor for asynchronous release, with the extent of this release modulated by the copy number ratio between fast binding, but low affinity calcium sensors such as Syt1 and slower, higher affinity sensors such as Syt7 (Maximov et al., 2008; Bacaj et al., 2013; Weber et al., 2014; Luo et al., 2015; Li et al., 2017).

Syt7 has also been linked to SV endocytosis. Early studies revealed that Syt7 overexpression increased the number of presynaptic endosomes (Virmani et al., 2003), with subsequent studies reporting slowed SV endocytosis in neurons overexpressing the sensor (Li et al., 2017). Interestingly, Syt7 knockdown was sufficient to restore normal SV endocytosis kinetics in Syt1 knockdown neurons, again suggesting a functional link between the two calcium sensors. These results suggest that Syt7 might facilitate endosomal recycling pathways such as ADBE. Syt7 trafficking is largely refractory to stimulation (Dean et al., 2012; Weber et al., 2014), in agreement with its predominant plasma membrane localization (Sugita et al., 2001; Maximov et al., 2008; Dean et al., 2012; Li et al., 2017). This atypical localization may explain some of the disparate functions ascribed to this calcium sensor. Recent evidence supports this view, with the plasma membrane localization of Syt7 critical for its control of multiple aspects of the SV life cycle (Vevea et al., 2021). In these studies, targeting Syt7 to either the plasma membrane or lysosome-associated membrane glycoprotein 1 (LAMP1)-positive vesicles, but not SVs, rescued the functional deficits observed in Syt7 knockout neurons. Plasma membrane targeting of Syt7 was dependent on its cleavage via γ-secretase and palmitoylation (Vevea et al., 2021), directly placing LEs and the endolysosomal system at the regulatory center of SV recycling.

The presence of two of the most abundant LE/lysosomal markers, VAMP7 and Syt7, in the SV pool suggests that lysosome-related organelles are intermingled in the pool of SVs, however, what could be their role? The evidence implicating LEs/lysosomes in regulation of local protein degradation is sparse. For example, LAMP1-containing organelles in distal axons have different acidity from the LAMP1-containing organelles in proximal axons indicating that they might have lower degradative capacity (Overly and Hollenbeck, 1996; Lie et al., 2021). In contrast, the ESCRT machinery is implicated in activity-dependent degradation of a subset of SV proteins via a pathway that requires Rab35 (Sheehan et al., 2016). Furthermore, cathepsins were detected in distal axons in the *Drosophila* brain and two parallel pathways, a Rab7-independent and a Rab7-dependent pathway were shown to specifically mediate synaptic degradation of SV proteins and membrane proteins, respectively (Jin et al., 2018). One study described a selective localization of the lysosomal protease cathepsin D to GABA-ergic presynapses and implicated it in the control of endocytic trafficking and GABA-ergic neurotransmission (Li et al., 2019). However, an argument against differential distribution of this lysosomal enzyme to inhibitory presynapses is the observation that cathepsin D knockout mice have global deficits in presynaptic ultrastructure (Partanen et al., 2008; Koch S. et al., 2011) and markedly decreased frequency of miniature excitatory postsynaptic currents, mEPSCs (Koch S. et al., 2011). Therefore, cathepsin D-containing lysosome-related organelles are present at both glutamatergic and GABA-ergic synapses and contribute to excitatory and inhibitory neurotransmission.

However, whether there are lysosomes with degradative capacity present at mammalian presynaptic terminals and what their contribution is, if any, to local degradation is currently unknown.

## Autophagy Function and Synaptic Vesicle Recycling

Another key axonal retrograde membrane trafficking route, fully integrated in the endolysosomal system, is the autophagy pathway. The generation of autophagosomes in synaptic terminals involves the sequential recruitment and activation of a series of protein complexes, with the main membrane donor being the endoplasmic reticulum [summarized in Kuijpers et al. (2021a), Overhoff et al. (2021)]. Indeed, when autophagy is disrupted via the loss of the key molecule autophagy protein 5 (Atg5), there is increased calcium release from presynaptic ER, resulting in enhanced excitatory neurotransmission (Kuijpers et al., 2021b). Synaptic autophagosome formation appears to be a constitutive process, unlike nutrient starvation-triggered events in non-neuronal cells (Maday and Holzbaur, 2014) and may aid the turnover/degradation of SVs and their cargos (Ravikumar et al., 2010; Maday et al., 2012). Maturation of autophagosomes into autolysosomes involves their fusion with degradative lysosomes and retrograde transport toward the cell body (Itakura et al., 2012; Takáts et al., 2013). Alternatively, they can also fuse with LE to form amphisomes, which perform discrete signaling roles, particularly when transporting growth factor receptors (Villarroel-Campos et al., 2018; Andres-Alonso et al., 2019). As outlined above, manipulation of the autophagy pathway results in altered neurotransmission, implicating this pathway as another control point in the SV life cycle. Furthermore, autophagy also requires proteins with defined roles in SV recycling and clathrin-independent endocytosis pathways (Milosevic et al., 2011; Pechstein et al., 2015; Kroll et al., 2019), such as endophilin and synaptojanin-1 (Soukup et al., 2016; Soukup and Verstreken, 2017; Vanhauwaert et al., 2017). In addition, the active zone protein bassoon modulates autophagy via sequestration of the Atg5 (Okerlund et al., 2017). This suggests that both SV recycling and autophagy pathways are intricately linked in terms of both function and molecular requirements.

## Discussion

The question whether SV recycling involves an endosomal intermediate is surprisingly still a matter of debate (Jähne et al., 2015), even when one considers that most, if not all, reported EE, RE, and LE markers are present in the SV pool and with many having direct effects on presynaptic function. Furthermore, the ever-increasing subdivision of functional SV pools described above suggests that the presynapse harbors a collection of functionally diverse organelles with distinct molecular compositions, not all of which engage directly in neurotransmission. This leads to the question, how inter-dependent are SV recycling and the endolysosomal system, and perhaps more provocatively, are these apparently different processes part of the same cellular continuum? In the section below, we outline a series of hypotheses, supported by the current literature which provide a potentially unifying model for membrane trafficking at the presynapse.

### How Homogenous Are Synaptic Vesicles and Are Many Recycling Endosomes/Late Endosomes in Disguise?

REs are a heterogeneous population of tubulovesicular membrane-bound organelles ∼60–100 nm in diameter (Willingham and Pastan, 1980; Willingham et al., 1984). An argument against a potential link between REs and SVs is the morphological uniformity of SVs that has been observed in multiple studies and attributed to the functioning of specific endocytic mechanisms (Zhang et al., 1998; Shimizu et al., 2003; Koo et al., 2015; Ivanova et al., 2020). However, electron microscopy (EM) studies from ultrathin sections have shown that the size of SVs exists in a range from 25 to 80 nm (Fox, 1988; Harris and Sultan, 1995). It is not always possible to reconstruct membrane continuity from such ultrathin sections and therefore the length at which SVs extend is difficult to appreciate. Contrary to the prevailing view that SVs have only a spherical shape, studies exist showing that SVs may not be morphologically uniform. Pleomorphic vesicles have been described in early EM studies, mostly as a marker for inhibitory synapses (Uchizono, 1965 and more recently, Koo et al., 2015; Li et al., 2019). With the advent of new technologies (e.g., cryo-EM and electron tomography) there is increasing evidence that tubule-shaped ellipsoidal vesicles are present at both excitatory and inhibitory synapses (Tao et al., 2018). Therefore, similarly to REs, SVs show a degree of morphological heterogeneity, with a diameter that ranges within tens of nanometers.

**FIGURE 1:**
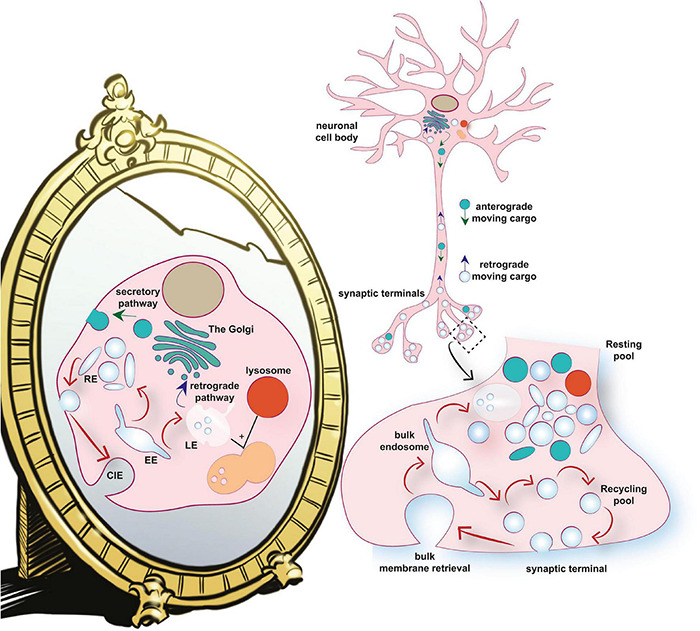
The SV life cycle as a mirror image of the general endolysosomal system. **(A)** The endolysosomal system consists of a set of membrane-bound compartments that undergo dynamic interconversion. At the center of this system is the Golgi apparatus, which is a steady-state organelle that apart from a central sorting hub in the secretory pathway, functions as an important quality control checkpoint that constantly receives cargo from the plasma membrane and the peripheral endosomes. The endolysosomal system is comprised of: (1). Early endosomes (EEs). EEs are organelles that receive membrane cargos and solutes from the extracellular environment through endocytosis. The main mode of endocytosis in most cells is clathrin-independent endocytosis (CIE). The EE sorts these cargos into recycling or degradative compartments of the cell. (2). Recycling endosomes (REs). REs are compartments involved in the slow constitutive and regulated recycling of cargo to the plasma membrane. (3). Late endosomes (LEs). The central role of LEs is sorting of ubiquitinated membrane proteins into intralumenal vesicles which form multi vesicular bodies (MVBs). MVBs fuse with lysosomes to form a degradative compartment in which protein cargos and intralumenal vesicles are degraded. LEs are also involved in the retrograde retrieval of cargo molecules from the plasma membrane and other endosomes to the Golgi apparatus. (4). Lysosomes are membrane-bound organelles that degrade and recycle cellular waste. In addition, they play an important role in cellular signaling and energy metabolism. **(B)** The dominant endocytosis modes operating at the central nerve terminals (ADBE and ultrafast endocytosis) are CIE pathways. The bulk endosome is emerging as a central sorting station, which similarly to the EE, sorts cargos to either the Recycling pool or the Resting pool. The Recycling pool (similarly to the REs) is implicated in the constitutive (spontaneous) and regulated (evoked) recycling of cargos to the plasma membrane. The Resting pool is likely a collection of functionally and molecularly heterogeneous membrane-bound organelles, which likely includes endolysosomal intermediates such as LE and lysosomes, which may or may not have a full degradative capacity. The membrane-bound organelles at the synapse are connected to endolysosomal organelles in neuronal cell bodies (the Golgi apparatus and the degradative lysosome) through antero- and retrograde-trafficking along the axon.

SVs are also functionally diverse and molecularly heterogeneous. Previous bulk biochemical approaches described the protein composition of a prototypical SV (Takamori et al., 2006; Wilhelm et al., 2014; Wittig et al., 2021). While providing insight into the molecular composition of an average SV, an important caveat of these bulk proteomic studies is that the cellular fractions that were analyzed represent averages of a diverse array of synapses and SVs. The copy number of common SV proteins, such as synaptobrevin2, synaptophysin, and synaptogyrin, in individual SVs shows a significant inter-vesicle variability, as revealed by single molecular quantification approaches (Mutch et al., 2011). Another important limitation of these early proteomic studies is that they have a bias toward proteins with high abundance, while underrepresented proteins with essential functions, often remain undetected. More recent studies report a longer list of proteins found in isolated synaptosomes, with low abundance proteins (less than 1 copy per SV) being the dominant fraction (Taoufiq et al., 2020). All of this suggests that different proteins are likely differentially distributed to different SV subpopulations. The presence of a multitude of endolysosomal molecules in the total SV pool that exert fine control over different steps of the SV cycle therefore strongly supports the notion that the two systems form a continuum (Figure 1).

The disparity of endolysosomal molecules within the SV pool (Table 1) suggests that some vesicles previously assumed to be SVs may in fact be intermediates of the endolysosomal pathway. In support, SVs are functionally similar to REs, as they are involved in slow, regulated and constitutive recycling of SV cargo to the plasma membrane. Furthermore, a handful of molecular markers characteristic for RE have been implicated in SV function as discussed above. For instance, the Rab35/Arf6 axis directly impacts SV composition and function. Furthermore the RE marker, Rab11 impacts calcium-regulated exocytosis of dense core vesicles and SVs and integrates regulated and constitutive exocytosis in neurons and neuroendocrine cells (Khvotchev et al., 2003), while facilitating SV endocytosis modes in central nerve terminals (Kokotos et al., 2018). The only described R-SNARE associated with RE, cellubrevin, is a structural homologue of the most abundant SV R-SNARE, synaptobrevin2 (McMahon et al., 1993). It quantitatively co-isolates with synaptophysin, when expressed in a heterologous system, suggesting that both localize on the same vesicles (McMahon et al., 1993). In further support of the hypothesis that some recycling SVs may be the equivalent of the RE in the presynaptic terminal, cellubrevin fully rescues synaptic transmission when expressed in synaptobrevin2-deficient neurons (Deák et al., 2006).

**TABLE 1:** Common endolysosomal proteins present in the SV proteome.

Protein	SV recycling role	Endolysosomal role	Presence on SVs
Syntaxin-6	Activity-dependent trafficking to plasma membrane (Hoopmann et al., 2010)	Endosome fusion (Maxfield and McGraw, 2004; Grant and Donaldson, 2009)	Yes (Takamori et al., 2006)
Syntaxin-7	Not determined	LE fusion (Luzio et al., 2010)	Yes (Takamori et al., 2006; Taoufiq et al., 2020)
Syntaxin-8	Not determined	LE fusion (Luzio et al., 2010)	Yes (Taoufiq et al., 2020)
Syntaxin-12	Activity-dependent trafficking to plasma membrane (Hoopmann et al., 2010)	Endosome fusion (Grant and Donaldson, 2009)	Yes (Takamori et al., 2006; Taoufiq et al., 2020)
Rab-4	Not determined	Axonal transport (Stenmark, 2009)	Yes (Takamori et al., 2006; Pavlos et al., 2010; Taoufiq et al., 2020)
Rab-5	Required for efficient SV recycling/SV cargo processing (Shimizu et al., 2003; Wucherpfennig et al., 2003; Star et al., 2005; Shin et al., 2008)	EE fusion/maturation (Stenmark, 2009)	Yes (Takamori et al., 2006; Pavlos et al., 2010; Taoufiq et al., 2020)
Rab-7	Not determined	EE to LE maturation (Stenmark, 2009)	Yes (Takamori et al., 2006; Pavlos et al., 2010; Taoufiq et al., 2020)
		LE fusion (Luzio et al., 2010)	
Rab-8	Not determined	Axonal transport (Stenmark, 2009)	Yes (Takamori et al., 2006; Pavlos et al., 2010; Taoufiq et al., 2020)
Rab-10	LDCV secretion (Sasidharan et al., 2012)	TGN to plasma membrane transport (Stenmark, 2009)	Yes (Takamori et al., 2006; Pavlos et al., 2010)
Rab-11	Facilitation of SV endocytosis/recycling (Steinert et al., 2012; Han et al., 2017; Kokotos et al., 2018)	RE maturation (Grant and Donaldson, 2009; Stenmark, 2009)	Yes (Takamori et al., 2006; Pavlos et al., 2010; Taoufiq et al., 2020)
Rab-14	Not determined	TGN to EE transport (Stenmark, 2009)	Yes (Takamori et al., 2006; Pavlos et al., 2010)
Rab-21	Not determined	EE to LE transport (Stenmark, 2009)	Yes (Takamori et al., 2006; Pavlos et al., 2010; Taoufiq et al., 2020)
Rab-35	Required for efficient SV recycling/SV cargo processing (Uytterhoeven et al., 2011; Sheehan et al., 2016)	RE fusion/maturation (Grant and Donaldson, 2009; Stenmark, 2009; Sheehan and Waites, 2019)	Yes (Takamori et al., 2006; Pavlos et al., 2010; Taoufiq et al., 2020)
EHD1	Required for ADBE (Jakobsson et al., 2011)	EE to RE transport (Grant and Donaldson, 2009; Naslavsky and Caplan, 2011)	Yes (Taoufiq et al., 2020)
Vti1a	Spontaneous SV fusion (Ramirez et al., 2012)	Endosome fusion (Maxfield and McGraw, 2004)	Yes (Takamori et al., 2006; Taoufiq et al., 2020)
	Activity-dependent trafficking to plasma membrane (Hoopmann et al., 2010)		
	SV fusion via TGN cargo trafficking (Emperador-Melero et al., 2018)	Retrograde TGN transport (Johannes and Popoff, 2008)	
Vti1b	SV fusion via TGN cargo trafficking (Emperador-Melero et al., 2018)	LE/lysosome fusion (Luzio et al., 2010)	Yes (Taoufiq et al., 2020)
AP1	Control of SV composition via SV cargo clearance (Candiello et al., 2016; Ivanova et al., 2021)	Cargo selection for retrograde transport (Grant and Donaldson, 2009)	Yes (Takamori et al., 2006)
VPS26	Not determined	Retromer cargo selection (Seaman, 2012; Burd and Cullen, 2014)	No
VPS29	Not determined	Retromer cargo selection (Seaman, 2012; Burd and Cullen, 2014)	Yes (Taoufiq et al., 2020)
VPS35	Facilitation of SV recycling (Inoshita et al., 2017)	Retromer cargo selection (Seaman, 2012; Burd and Cullen, 2014)	No
VPS34	Bulk endosome cargo sorting (Candiello et al., 2016)	Endosome to TGN transport (Grant and Donaldson, 2009)	No
SNX1	Not determined	Retromer tubulation (Seaman, 2012; Burd and Cullen, 2014)	Yes (Taoufiq et al., 2020)
SNX5	Not determined	Retromer tubulation (Seaman, 2012; Burd and Cullen, 2014)	Yes (Takamori et al., 2006; Taoufiq et al., 2020)
SNX6	Not determined	Retromer tubulation (Seaman, 2012; Burd and Cullen, 2014)	Yes (Taoufiq et al., 2020)
Arf6	Repression of SV recycling/SV cargo processing (Allaire et al., 2006)	RE maturation (Grant and Donaldson, 2009; Sheehan and Waites, 2019)	Yes (Taoufiq et al., 2020)
TBC1D24	Required for efficient SV recycling/SV cargo processing (Uytterhoeven et al., 2011; Fernandes et al., 2014; Finelli et al., 2019)	Rab35 GTPase activating protein (Uytterhoeven et al., 2011)	No
		Control of Arf6 activity (Falace et al., 2014; Aprile et al., 2019)	
Syt7	RRP Replenishment (Liu et al., 2014)	LE/lysosome fusion (Martinez et al., 2000; Arantes and Andrews, 2006)	Yes (Taoufiq et al., 2020)
	Asynchronous release (Maximov et al., 2008; Bacaj et al., 2013; Weber et al., 2014; Luo and Südhof, 2017)		
	Spontaneous release (Chanaday et al., 2021)		
	Facilitation of ADBE (Virmani et al., 2003; Li et al., 2017)		
VAMP3	Not determined	RE fusion (Grant and Donaldson, 2009)	Yes (Takamori et al., 2006; Taoufiq et al., 2020)
VAMP4	Control of Pr (Ivanova et al., 2021)	Endosome fusion (Maxfield and McGraw, 2004)	Yes (Takamori et al., 2006; Taoufiq et al., 2020)
	Required for ADBE (Nicholson-Fish et al., 2015; Ivanova et al., 2021)		
	Asynchronous release (Raingo et al., 2012)	Retrograde TGN transport (Johannes and Popoff, 2008)	
	Spontaneous release (Raingo et al., 2012; Lin et al., 2020)		
VAMP7	Spontaneous SV fusion (Hua et al., 2011; Ramirez et al., 2012; Bal et al., 2013)	LE/lysosome fusion (Rao et al., 2004; Arantes and Andrews, 2006; Luzio et al., 2010)	Yes (Takamori et al., 2006; Taoufiq et al., 2020)
ATG5	Not determined	Presynaptic autophagy (Kuijpers et al., 2021a; Overhoff et al., 2021)	No
Endophilin	Required for SV uncoating (Gad et al., 2000; Milosevic et al., 2011; Pechstein et al., 2015)	Presynaptic autophagy (Soukup et al., 2016; Soukup and Verstreken, 2017)	Yes (Taoufiq et al., 2020)
	Required for SV endocytosis (Gad et al., 2000; Sundborger et al., 2011; Watanabe et al., 2018)		
	Calcium influx and SV recycling (Kroll et al., 2019; Gowrisankaran et al., 2020)		
LAMP1	Not determined	Maintenance of lysosome integrity (Saftig and Klumperman, 2009)	Yes (Takamori et al., 2006; Taoufiq et al., 2020)

*Many endolysosomal proteins are part of the cohort of molecules present in highly purified SV fractions. This table reports common endolysosomal molecules, their proposed role in the SV life cycle and their presence on purified SVs.*

Similarly, LE/lysosome fusion events may be responsible for a series of functional outputs ascribed to particular SV subpopulations. The mobilization of the resting SV pool is of particular interest, since the LE/lysosome effectors VAMP7 and Syt7 are both represented in this SV pool. For example, the calcium-dependent nature of the VAMP7-dependent fusion events evoked by reelin (Bal et al., 2013), is reminiscent of ionophore-evoked lysosome fusion in non-neuronal cells. This may have great relevance, since the plasma membrane deposition of Syt7 impacts a series of SV recycling events (Vevea et al., 2021), suggesting lysosome fusion is a prerequisite for optimal presynaptic function. However, the reciprocal control of the resting SV pool by the calcium-activated protein phosphatase calcineurin and the protein kinase cyclin-dependent kinase 5 (Kim and Ryan, 2010) appears to operate in a reverse manner for lysosome fusion (Medina et al., 2015; Ishii et al., 2019). Therefore, more work is required to determine the specific molecular nature of the organelles that undergo regulated fusion at the presynapse.

### A Reappraisal of the Synaptic Vesicle Pool Nomenclature

The concept of SV pools was introduced as a model to explain the functional diversity of SVs present in the presynaptic terminal and their distinct contribution to different forms of neurotransmitter release (Alabi and Tsien, 2012). As hypothesized above, the ever-increasing complexity of SV subpopulations that contribute to specific functional fusion or release events suggests a disparate composition of the total SV pool, with potentially direct contributions from different endolysosomal organelles. The SV pool model has proven its utility for describing the plasticity of neurotransmitter release in terms of changes of SV pool sizes (Kim and Ryan, 2010; Rey et al., 2020). However, as with any model, it has its limitations (Neher, 2015). The main limitation is that it remains too deterministic in its original interpretation, and in specific cases, it postulates that distinct pools with specific molecular composition mediate different forms of release (Crawford and Kavalali, 2015). In reality, the dynamic behavior of complex biological systems, such as the SV life cycle, is inherently stochastic. Molecules are randomly distributed to different SV populations and complex regulatory mechanisms guide probabilistic outcomes. Because of this, qualitatively similar functional outcomes may arise from the interactions of different and varied number of molecules, each present on SVs in a small absolute quantity.

The pool model also fails to explain why the resting pool both *in vitro* and *in vivo* cannot be released even after extensive periods of synaptic stimulation (Alabi and Tsien, 2012). The reluctant nature of these SVs to participate in activity-dependent recycling suggest that they might be endosomes that have roles other than a direct contribution to neurotransmitter release. For instance, these resting SVs might function as a reservoir of molecules that can be incorporated into the recycling pool on demand (during sorting, as discussed below) to allow plastic changes of SV composition and function (Denker et al., 2011). In addition, part of the resting pool, may in fact be comprised of a series of endolysosomal intermediates primed for transport to neuronal cell bodies and the degradative pathways (Figure 1).

In many cases, the pool model comes short in providing a good correlation between morphology and function. Thus, loss of function of molecules, such as synaptobrevin2 (Deák et al., 2004), munc-13 (Varoqueaux et al., 2002), munc-18 (Verhage et al., 2000), and calcium channels (Held et al., 2020), that strongly reduce or eliminate any forms of neurotransmitter release, does not have any major impact on synaptic ultrastructure and the number of SVs per synaptic terminal. In contrast, the integrity of the SV cluster is compromised when the function of key endosomal sorting proteins is abrogated (Glyvuk et al., 2010; Tagliatti et al., 2016). This implies that the SV pool and its clustering at the synapse are driven by intrinsic programs in the neuron and SV recycling is not essential for maintaining its integrity.

Contrary to the pool model, SV fusion and neurotransmitter release are likely an emerging property of a small subset of the endolysosomes orchestrated in the presynaptic terminal that has been selected through evolution to support the central function of the nervous system, neuronal communication. Indeed, the ultrastructural organization of excitatory presynaptic terminals that developed in complete absence of glutamate release (due to Cre-induced expression of tetanus toxin), was largely preserved, with a normal number of SVs and led to normal refinement of connectivity in the developing brain (Sando et al., 2017; Sigler et al., 2017). Furthermore, the universal excitatory (glutamate, aspartate, and cysteic acid) and inhibitory neurotransmitters (GABA, glycine), are amino acids and the endolysosomal system is a well-established storage site for free amino acids (Russnak et al., 2001; Abu-Remaileh et al., 2017). Some of these intralumenal amino acids are a key element of a central endolysosomal mechanism for nutrient sensing and metabolic control that operates in virtually all cells and organs (Lawrence and Zoncu, 2019). Furthermore, the transport of classical neurotransmitters and neuromodulators into SVs is driven by members of the solute carrier family of transporters (SLC) (Schuldiner et al., 1995; Anne and Gasnier, 2014), that are broadly expressed in various endolysosomal structures and are essential to vital processes within and outside the nervous system (Serrano-Saiz et al., 2020). In addition to their ability to transport amino acids, some of these channels operate as ion channels and regulate the ion gradients and acidification of endosomes (Martineau et al., 2017). The import of neurotransmitters could therefore be considered a by-product of the establishment of these electrochemical gradients, that have additional roles in the functioning of endosomes.

Additional support for the hypothesis that the SV pool is not an autonomous module, operating independently in isolated synaptic terminals, is provided by the existence of a superpool of SVs. Although it is generally accepted that the SV pool and recycling are local, synapse-specific phenomena, overwhelming evidence exists that the pool is not confined to a particular synapse, but spans multiple synapses in a single axon (Staras et al., 2010). Photobleaching and photoactivation experiments have demonstrated that recycling SVs, and synaptic proteins in general, quickly redistribute among neighboring synapses and even between synapses and neuronal cell bodies (Tsuriel et al., 2006; Staras et al., 2010; Ivanova et al., 2015). Thus, the SV pool is shared among many synapses and consists of multiple interconnected organelles that continuously shuttle (in both directions) between axons and neuronal cell bodies. Based on this, a different model emerges, in which the SV pool can be presented as a collection of molecularly heterogeneous endosomes. Owing to the accumulation of specific sets of molecules during the elaborate process of endosomal sorting, a small fraction of these endosomes engages in regulated (evoked) as well as constitutive (spontaneous) release in the confines of a specific synapse (Kaeser and Regehr, 2014; Chanaday and Kavalali, 2018). However, the SV cycle is nested in a larger cycle that encompasses membrane-bound organelles that reside in both, presynaptic sites and neuronal cell bodies.

### Lessons Learned From Studying the Trafficking of the Endosomal R-SNARE VAMP4 at the Presynapse

As described above, VAMP4 is an endosomal R-SNARE implicated in homo- and heterotypic endosomal fusion which continuously shuttles between endosomes and the TGN in non-neuronal cells. In neurons it is enriched at the TGN as well as in distal axons. In nerve terminals, the majority of VAMP4 is sorted during ADBE to vesicles that are refractory to synaptic stimulation (Nicholson-Fish et al., 2015; Ivanova et al., 2021). Retrograde axonal trafficking of VAMP4 on vesicles that are positive for Rab7 (presumably LEs) mediates its continuous retrieval from nerve terminals and recycles it back to neuronal cell bodies. A small fraction of the VAMP4 pool is present on SVs that undergo activity-dependent exocytosis (recycling SVs) and its abundance in the SV pool inversely correlates with SV fusion (Ivanova et al., 2021). Thus, VAMP4 is an example of a protein that shows a wide distribution to different membrane-bound organelles, once again highlighting the interconnectedness of the endolysosomal system and SVs.

Activity-dependent SV endocytosis modes such as ultrafast endocytosis and ADBE are the dominant mechanism for SV cargo and membrane retrieval at central synaptic terminals (Chanaday et al., 2019). Therefore, the great majority of SVs mobilized during synaptic activity recycles through a common endosomal intermediate, the bulk endosome. To mediate efficient neurotransmitter release, recycling SVs must garner a specific set of proteins in a certain stoichiometric ratio. However, a systematic model of the molecular mechanisms underpinning differential protein sorting during SV recycling is currently missing. Di-leucine and tyrosine-based motifs are known to be involved in endolysosomal targeting of proteins (Bonifacino and Traub, 2003), such as with VAMP4, whose cytoplasmic domain possesses a di-leucine motif which mediates its AP1-dependent sorting to endosomes and lysosome-related organelles (Peden et al., 2001). The presence of a di-leucine signal in VAMP4 is likely the underlying reason for its limited localization to recycling SVs and its enrichment on bulk endosomes (Nicholson-Fish et al., 2015).

However, there is no common sorting motif responsible for selective targeting of proteins to the recycling SV pool. This might indicate the existence of distinct sorting mechanisms for each individual SV cargo. An alternative model stipulates that removal of molecules is more important than selective incorporation of proteins for generating of SVs with an optimal complement of proteins for recycling. According to this model, recycling SVs, exactly like REs, are the remnants of bulk endosomes (the equivalent of EE) that persist after cargo sorting to LE. The incorporation of classic endolysosomal proteins, such as VAMP4, in the SV pool makes endolysomal sorting a prerequisite step in the reformation of SVs. Based on the similarity between EEs and synaptic bulk endosomes, it is easy to envisage that following endocytic retrieval, the newly formed bulk endosomes mature to a dynamic sorting compartment by undergoing heterotypic fusion with pre-existing vesicles/endolysosomes from the resting SV pool. This hypothesis is supported by *in vitro* data showing that newly endocytosed SVs undergo homotypic fusion and are capable of fusing with early endosomes isolated from other cell types (Rizzoli et al., 2006). However, despite the increasing level of innovation in the imaging tools that drive rapid advancement in cellular neurobiology, currently there is no *in cellulo* data supporting endosomal fusion at the presynapse. More than anything else, this indicates the urgent need for developing of sufficiently sensitive techniques that break the diffraction barrier but simultaneously allow real-time visualization of membrane trafficking in the confinements of the conventional chemical synapse.

Despite these currently insurmountable technical limitations, we recently showed (indirectly, through studying VAMP4 trafficking at the presynapse) that intermixing of cargos retrieved through endocytosis with classic endolysosomal cargos, presumably prior to endolysosomal sorting, can introduce molecular heterogeneity in SV composition and drive plasticity of neurotransmitter release (Ivanova et al., 2021). This attunes synaptic transmission to both the history of presynaptic activity, and the functionality status of the quality control mechanisms operating throughout the endolysosomal system. More importantly, endolysosomal sorting during SV recycling provides a potential conduit for a constitutive, use-dependent turnover of SV components. In support, blocking the sorting of VAMP4 to LEs, using a dominant negative form of Rab7, increased its synaptic expression and slowed down its synaptic turnover (Ivanova et al., 2021). Interestingly, the same intervention also increased the synaptic expression of synaptophysin, although to a lesser extent. Therefore, it is tempting to speculate that active sorting of classic endolysosomal proteins, such as VAMP4, to LEs may promote passive retrieval of SV cargos that do not necessarily possess sorting determinants for targeting to LEs/lysosomes. This can provide a stochastic, pre-emptive mechanism for removal of SV cargos that have participated in recycling (that has been speculated to exist Truckenbrodt et al., 2018) which continuously rejuvenates the SV proteome and thereby maintains synaptic integrity and function.

### Cycling Back to Neuronal Cell Bodies; Potential Impact of Re-trafficking to the Trans-Golgi Network and Somatic Lysosomes on Presynaptic Function, Lessons Learned From Models of Disease

The rapid redistribution of SV proteins between neighboring synapses (in a matter of minutes; Tsuriel et al., 2006; Staras et al., 2010) and between synapses and neuronal cell bodies (in a matter of hours Tsuriel et al., 2006; Ivanova et al., 2015) observed using various live-imaging approaches, indicates a very rapid axonal turnover of SV components. However, this is at odds with the very slow metabolic turnover of SV proteins, which ranges from several days to several weeks *in vitro* and *in vivo*, respectively (Dorrbaum et al., 2018). This suggests that SV proteins may undergo multiple rounds of trafficking between synapses and neuronal cell bodies before being directed to any of the degradative routes. Although synapses are located at a considerable distance from neuronal cell bodies, owing to the function of motor proteins the communication between them is a very rapid and efficient process (Guedes-Dias and Holzbaur, 2019). However, why would neurons support such a metabolically-expensive strategy to continuously shuttle vesicles and molecules between distal synapses and neuronal cell bodies?

**FIGURE 2:**
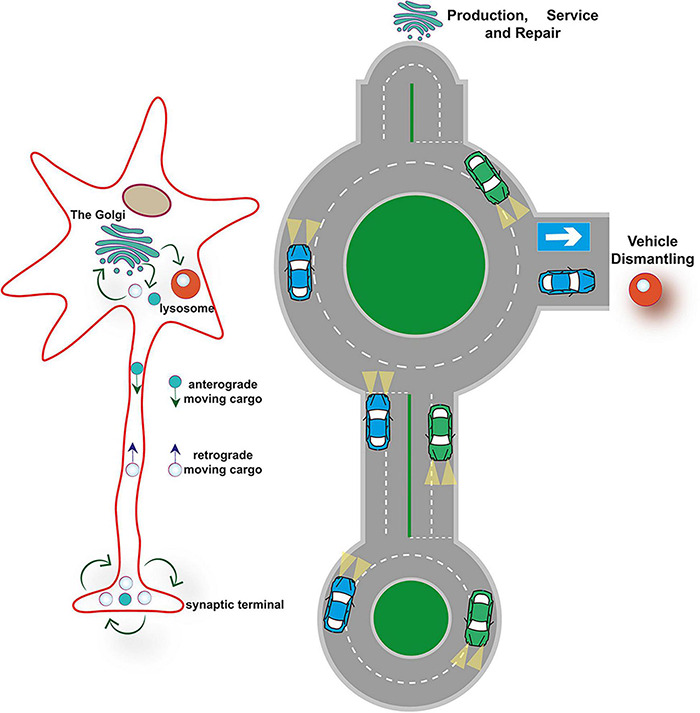
SV recycling is nested in the endolysosomal system. SV recycling is a nested cycle within a larger cycle that encompasses membrane-bound organelles that reside in both, presynaptic sites (the Recycling and Resting pool) and neuronal cell bodies (the Golgi apparatus and degradative lysosomes). The Golgi apparatus performs protein surveillance and funnels cargos to pathways that either repair damage or degrade old and damaged proteins (the lysosome).

The TGN, which is a central sorting hub in the secretory pathway, is also emerging as a major protein quality control checkpoint (Briant et al., 2017; Hellerschmied et al., 2019; Sun and Brodsky, 2019). Similarly to the quality control systems operating in the ER, specialized molecular machineries residing in the TGN perform protein surveillance and funnel damaged, misfolded or aggregated proteins into pathways that either attempt repair or sequester and degrade the damaged proteins (typically through lysosomal degradation) (Sun and Brodsky, 2019). Therefore, continuous retrograde trafficking and recycling through the TGN of plasma membrane proteins and proteins that normally localize to other endolysosomal compartments, will allow a periodical reevaluation of their state (Figure 2).

This hypothesis that recycling of synaptic proteins to the TGN is essential for synapse integrity, is strongly supported by the key role of retromer in maintaining synaptic health and its involvement in the progression of neurodegenerative diseases such as Alzheimer’s and Parkinson’s disease (Small and Petsko, 2015). Multiple genetic studies have linked both Alzheimer’s and Parkinson’s disease to a number of retromer-associated proteins and enhancement of the retromer function was neuroprotective against the pathology of these slowly progressing neurodegenerative diseases (Vilariño-Güell et al., 2011; Wen et al., 2011; Small and Petsko, 2015; McMillan et al., 2017; Brodin and Shupliakov, 2018). The neuroprotective role of the retromer complex is believed to be coupled to its role in retrieving cargo from the plasma membrane and the endosome back to the TGN. However, whether the retrograde trafficking from synaptic endosomes to the TGN is limited to specific cargos or is a more widespread phenomenon with a major impact on synaptic function, remains an open question.

In the light of this hypothesis, one possible function for presynaptic LEs/lysosomes is sorting of SV cargos during SV recycling and directing them to trafficking pathways that carry them back to the soma where they can be subjected to quality control sampling (at the TGN) and/or degradation by somatic lysosomes. This is supported by the clear functional link between lysosomal function and presynaptic health. Neurodegenerative processes associated with lysosomal dysfunction, as in lysosomal storage diseases (LSD), are usually presynaptically initiated and neurodegeneration in these conditions can be curbed by re-establishing presynaptic function (Sambri et al., 2017). SV exo- and endocytosis are severely compromised by lysosomal storage dysfunction. Thus, in several LSD mouse models (Twitcher mice, Niemann-Pick disease type C1, and Mucopolysaccharidosis type 3A), axonal transport of SV-positive vesicles, SV exo-and endocytosis are critically impaired (Xu et al., 2010; Pressey et al., 2012; Wilkinson et al., 2012; Teixeira et al., 2014; Sambri et al., 2017; Bayó-Puxan et al., 2018). This is associated with reduction or aggregation of SV proteins and axonal swelling which results in compromised neurotransmission. Some of these deficits can be partly attributed to interference with the function of presynaptic autophagy (and reduced fusion of autophagosomes and lysosomes). However, the severe presynaptic phenotypes in LSD mouse models, strongly suggest that the SV cycle is a loop within a loop, and that it is structurally and functionally integrated in the general endolysosomal system of the neuron. Therefore, deficits at either end of the system will compromise the function of the whole system.

### Outlook

Transitioning from a purely analytic approach to SV recycling to a more systemic one at a molecular level, which recognizes and studies the SV cycle as an integral part of the endomembrane system of the neuron is key for answering outstanding questions in the field by setting the right priorities for future research. Some of these questions are: Which are the cell-biological mechanisms underlying the assembly and removal of the presynaptic specializations? What drives the use-dependent structural and functional presynaptic plasticity that alters the information coding capacity of the neuron? What mediates the ongoing turnover of SV components which sustains synaptic integrity and function over the lifespan of the neuron? How does previous experience impact these events and is this process tuneable to external signals? Furthermore, multiple neurological disorders ranging from neurodevelopmental autism spectrum disorders and epilepsy to slowly progressing neurodegenerative disorders can be linked to deficits in endolysosomal function and concomitant presynaptic malfunction. To prevent or delay the onset of such disorders and assist in developing targeted therapies in the future, disentangling the systems that regulate these complex diseases is key and requires examining the interplay among the underlying factors at a molecular level.

## Author Contributions

DI conceived the topic. DI and MC wrote all drafts of the manuscript. Both authors contributed to the article and approved the submitted version.

## Conflict of Interest

The authors declare that the research was conducted in the absence of any commercial or financial relationships that could be construed as a potential conflict of interest.

## Publisher’s Note

All claims expressed in this article are solely those of the authors and do not necessarily represent those of their affiliated organizations, or those of the publisher, the editors and the reviewers. Any product that may be evaluated in this article, or claim that may be made by its manufacturer, is not guaranteed or endorsed by the publisher.
